# Surface Evaluation of Aligners after Immersion in Coca-Cola and Orange Juice

**DOI:** 10.3390/ma15186341

**Published:** 2022-09-13

**Authors:** Maciej Warnecki, Michał Sarul, Marcin Kozakiewicz, Anna Zięty, Bartosz Babiarczuk, Beata Kawala, Kamil Jurczyszyn

**Affiliations:** 1 Independent Researcher, Niemodlińska 63, 45-864 Opole, Poland; 2 Department of Integrated Dentistry, Wrocław Medical University, 50-425 Wrocław, Poland; 3 Department of Maxillofacial Surgery, Medical University of Lodz, 113 Żeromskiego Str., 90-549 Lodz, Poland; 4 Department of Mechanics, Materials and Biomedical Engineering, Faculty of Mechanical Engineering, Wrocław University of Science and Technology, Smoluchowskiego 25, 50-372 Wrocław, Poland; 5 Department of Dentofacial Orthopedics and Orthodontics, Wrocław Medical University, Krakowska 26, 50-425 Wrocław, Poland; 6 Department of Oral Surgery, Wrocław Medical University, Krakowska 26, 50-425 Wrocław, Poland

**Keywords:** orthodontic aligners, orthodontic aligner surface, fractal dimension analysis, texture analysis, wetting angle, Coca-Cola, orange juice

## Abstract

Orthodontic removable appliances made of transparent thermoplastic materials—aligners—are becoming increasingly popular in contemporary orthodontic practice. It is important for the clinician to fully understand the mechanical properties and behavior of the appliance used. Because of that, the aim of our study was to investigate the changes in aligner surface after immersion in Coca-Cola and orange juice. For surface evaluation, fractal analysis, texture analysis, and wetting angle measurement were performed. Statistically significant changes were found between some of the groups in the fractal dimension analysis. In texture analysis, all but one intergroup comparison showed statistically significant differences. For wetting angle assessment, statistically significant differences were found. These were, however, more numerous when assessing glycol droplets, rather than water droplets. Fractal dimension analysis confirmed a correlation between the intensity of changes in the aligner surface with immersion time in the liquids assessed. Texture analysis showed a high sensitivity to the changes in aligner surface. It failed, however, to reveal changes relative to immersion time. Wetting angle analysis revealed aligner surface degradation for Coca-Cola. It did not, however, prove the dependence of the intensity of this degradation as a function of time. Both Coca-Cola and orange juice can cause aligner surface degradation.

## 1. Introduction

Edgewise fixed orthodontic appliances have been in use for over 100 years. The contemporary standards with regard to aesthetic looks, also in terms of the appearance of teeth and smile, have resulted in the use of orthodontic therapy also in adult patients. The same standards make patients want their therapeutic appliances to be as little visible as possible. Traditional orthodontic brackets are often not considered sufficiently aesthetically pleasing and, as they promote the accumulation of dental plaque, they increase the risk of caries, gingivitis, and periodontitis [1]. Even though the use of elastic positioning appliances was described as early as 1945 [2], it is only for the above-mentioned reasons that invisible orthodontic aligners (IOAs) are increasingly gaining popularity. They are an innovative alternative for fixed orthodontic appliances due to their ability to be easily applied/removed from the mouth without affecting the ability to masticate or the aesthetic look of the patient’s smile [3]. 

The market has seen the emergence of a considerable number of companies dealing with aligners that employ a similar strategy in treating patients. Thermoplastic materials used by aligner manufacturers currently include polyethene terephthalate glycol-modified (PET-G), polypropylene, polycarbonate (PC), thermoplastic polyurethanes (TPUs), ethylene-vinyl acetate, and many others. Materials should be biocompatible, transparent, have a low level of toughness and high elasticity, and be able to effectively fix the alignment of teeth [4]. For this reason, many authors have undertaken the analysis of the mechanical and chemical properties of aligners [5,6,7,8,9]. 

It is recommended that a single aligner be used for a period of 14 days. During that period, the brace is exposed to the environment of the patient’s oral cavity, with highly variable temperature and pH conditions. Therefore, it is important to determine the influence of the ageing of these elements under the influence of the oral environment with regard to their mechanical properties [9]. The most frequently analyzed data include the elasticity coefficient and the surface topography of these elements [5,6,8,9,10]. The physical and chemical properties of the oral environment can affect the level of changes occurring in the structure of the brace material. Beverages such as Coca-Cola or fruit juices are one of the main factors affecting them [11,12]. 

Various methods of microscopic imaging (SEM, AFM), laser spectroscopy, and X-ray diffraction analysis are the techniques most often used to analyze the surface of orthodontic components. However, the use of such methods may require application of complicated and expensive equipment and does not always allow conclusions to be drawn about functional properties [13,14]. A relatively simple method based on image analysis is the analysis of the values of the fractal dimension and texture analysis. They are increasingly often applied for analyzing the surface of elements used in orthodontic therapy [15,16]. For assessing the level of surface degradation, wetting angle analysis can also be used. This method assesses the shape of droplets forming on the analyzed surface—the higher the wetting angle (higher hydrophobicity), the lower the wettability of the element, and thus the cleaner the surface, and the other way around—the smaller the angle, the higher the wettability. High surface energy of the material and low surface tension of the solution decrease the value of the wetting angle. The level and time in which dental/orthodontic biomaterials absorb moisture from the oral environment have a considerable impact on the durability of these elements and determine the level of their degradation [17].

The present study aimed to determine the changes in the surface properties of aligners under the influence of the immersion in Coca-Cola and fruit juice with the use of fractal dimension analysis, texture analysis, and wetting angle analysis.

A null hypothesis was proposed that immersion in orange juice and Coca-Cola does not cause changes to the surface properties of aligners, as measured with fractal dimension analysis (FD), texture analysis (TA), and wetting angle analysis (WA).

## 2. Materials and Methods

Aligners made of three-layer polyurethane (Zendura^TM^ FLX) were selected for the test. The material was 0.76 mm thick, consisting of two hard outer shells and an elastic inner core. All aligners were made using one selected model, only for the purpose of this experiment, increasing its range within the palate. A 25 × 25 mm aligner element was selected for the study, located on its palatal surface.

To assess the effect of immersing aligners in different beverages, a total of 75 aligner samples were selected. Each of them was initially molded on the same model.

Then, the aligners were divided into 5 groups:

1. The control group—not immersed—15 aligners;
2. A group immersed in Coca-Cola (pH = 2.5) for one week (C1W)—15 aligners;
3. A group immersed in Coca-Cola (pH = 2.5) for two weeks (C2W)—15 aligners;
4. A group immersed in orange juice (pH = 3.5) for one week (J1W)—15 aligners;
5. A group immersed in orange juice (pH = 3.5) for two weeks (J1W)—15 aligners.

The total size of the studied group of aligners was 75 specimens. 

The Coca-Cola beverage used was the Coca-Cola Original Taste^TM^ beverage, which is a trademark of The Coca-Cola Company. The orange juice beverage used was the “Tymbark^TM^ sok 100% pomarańcza”, which is a trademark of Tymbark-MWS Sp. z o.o., a popular brand of juice in Poland.

The immersion involved immersing aligners in a beverage 3 times a day for 1 h, at room temperature (22 °C), as follows:

- In freshly squeezed orange juice for groups J1W and J2W—for 1 or 2 weeks, respectively.
- In Coca-Cola for groups C1W and C2W—for 1 or 2 weeks, respectively.

After completion of the immersion, each piece was rinsed for 15 min with demineralized water, and in a dry state, they were submitted for testing within 24 h.

### 2.1. Taking Images

All images for analysis were taken using the scanning electron microscope (SEM)—VEGA3 (Tescan, Brno—Kohoutovice, Czech Republic). The resolution of images was 1280 × 1430, with a magnification of 1000×, a voltage of 15 kV, and a backscattered electron (BSE) detector. In every group of aligners, 15 regions of interest (ROIs) for fractal dimension and texture analyses were set. All ROIs were 25 mm × 25 mm in size. ROIs were saved as 8-bit grayscale bitmaps. To apply all necessary graphical operations, GIMP version 2.10.30 (GNU Image Manipulation program: www.gimp.org, free and open-source license, accessed on 1 April 2022) was used.

### 2.2. Fractal Dimension Analysis

All fractal analyses were performed in ImageJ, version 1.53e (Image Processing and Analysis in Java—Wayne Rasband and contributors, National Institutes of Health, USA, public domain license, https://imagej.nih.gov/ij/ (accessed on 1 April 2022)), and the FracLac plugin, version 2.5 (Charles Sturt University, Bathurst, Australia, public domain license). 

A modified algorithm of the box-counting method, which makes it possible to analyze monochromatic images (such as 8- or 16-bit images) was used in the presented analysis. For the greyscale images, the intensity difference algorithm was used to calculate the fractal dimension, as it was applied in other studies examining the surface of orthodontic components with the FDA method [16]. The analyzed image was divided into boxes, as in the box-counting method. The image size selected for analysis was 25 × 25 μm. Performing every FDA, repetitive actions were performed: in the first step, the grid size equals 200 mm (dimension of the analyzed image, ε = 1); then, ε is divided by 2 (ε value for the following steps: ε = 0.5; ε = 0.25). In each step, the difference in pixel brightness intensity is calculated in every grid on scale ε. In the FracLac plugin, the algorithm of the ε calculation is called a block series. This option scans a square block within an image using a series of grids calculated from the block size. According to the authors, this specific algorithm is the best solution when analyzing the pattern that fills the entire area of the image.

The difference between the maximum pixel intensity and the minimum pixel intensity is calculated in each box (δI_i,j,ε_, where i, j—the location of the analyzed box in the ε scale) [14,18]:

δI_i,j,ε_ = maximum pixel intensity_i,j,ε_ − minimum pixel intensity_i,j,ε_ (1)

In the next step, 1 is added to the intensity difference to prevent its value from becoming a 0 [14,18]:

I_i,j,ε_ = δI_i,j,ε_ + 1 (2)

Finally, the fractal dimension of the intensity difference is described using the following formula [14,18]:

(3) $$\mathrm{FD}\;=(\underset{\varepsilon \to 0}{\lim}\frac{\ln\left(I\varepsilon \right)}{\ln\left(\frac{1}{\varepsilon }\right)})$$

where FD is the final fractal dimension of intensity, Iε = Σ [1δI_i,j,ε_ + 1], and ε is the box scale.

### 2.3. Texture Analysis

SEM images of orthodontic aligner surfaces were evaluated in ROIs of 25 µm × 25 µm. The surface texture was evaluated using features derived from the co-occurrence matrix. The regions of interest (ROIs) were normalized (μ ± 3σ) to share the same average (μ) and standard deviation (σ) of optical density within the ROIs. Selected image texture features (entropy and difference entropy from the co-occurrence matrix and long-run emphasis moment from the run-length matrix) in ROIs were calculated for the reference bone and the bone with the collagen scaffold applied [19]:

(4) $$Entropy=-\sum_{i=1}^{Ng}\sum_{j=1}^{Ng}p\left(i,j\right)log\left(p\left(i,j\right)\right)$$

where Σ is the sum, *Ng* is the number of optical density levels in the radiograph, *i* and *j* are the optical density of pixels that are 5 pixels away from one another, *p* is probability, and log is the common logarithm. 

Calculations were performed in the MaZda 4.6 program [15,16,18,19,20,21,22,23,24]. Entropy values were compared between groups with the Kruskal–Wallis test. When *p* < 0.05, the difference was considered statistically significant. Statgraphics Centurion 18 ver.18.1.12 (StarPoint Technologies, Inc., Warrenton, VA, USA) was used for statistical analyses.

### 2.4. Wetting Angle Assessment

Two wetting agents were used to test the wetting angle. H_2_O was selected as the substance with polar properties. Ethylene glycol was chosen as a substance that does not have the properties of dissolving the aligner material and, at the same time, has nonpolar properties.

**Figure 1:**
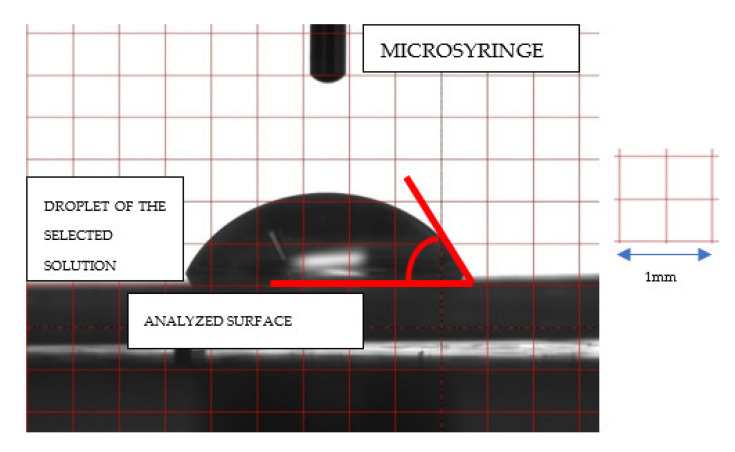
Illustrative image of a droplet on the surface of a sample, taken with a CCD camera.

In the presented test, droplets of approximately 3 μL of water or glycol were placed on the surface of the sample using a microsyringe, keeping the needle at the same minimum height above the surface to be analyzed, and maintaining the truncation direction of its tip. The droplet was photographed with a CCD camera (Hamamatsu ORCA-285G CCD Digital Camera C4742-95-12G04, Boston Industries) 5 s after it was placed on the sample surface. A minimum of six droplets were placed on each sample, each time on a ‘fresh’ surface—Figure 1.

### 2.5. Statistical Analysis

Statistica, version 13.3 (StatSoft, Cracow, Poland), was used to perform statistical tests in the aspect of fractal dimension analysis, wetting angle, and surface tension. A value of 0.05 was deemed to be statistically significant. The Shapiro–Wilk test was applied to confirm the normality of distribution. Due to the lack of a normal distribution, the nonparametric Kruskal–Wallis test was applied to reveal differences between groups. The Spearman coefficient was used to check for correlations between variables. Texture comparisons between wire sides and the material were performed with the one-way ANOVA or the Kruskal–Wallis test, depending on the presence of a normal distribution. When *p* < 0.05, the difference was considered statistically significant. The assumed power of the test was greater than 0.8. Statgraphics Centurion 18, version 18.1.12 (StarPoint Technologies, Inc., Warrenton, VA, USA), was used for statistical analyses.

## 3. Results

### 3.1. Fractal Dimension Analysis

Table 1 shows the results of statistical differences in fractal dimension (FD) values between the different series of samples. Statistically significant differences of the control group with the J2W and C2W groups, and of the J1W group with the J2W and C2W groups were found. As the only group, the C1W group showed no differences compared to any other group.

The results of the correlation of FD and TA with wetting angle, surface tension for water and glycol, and total tension are presented in Table 2. There was no correlation between FD and wetting angle with water and glycol or total tension.

### 3.2. Texture Analysis

**Figure 2:**
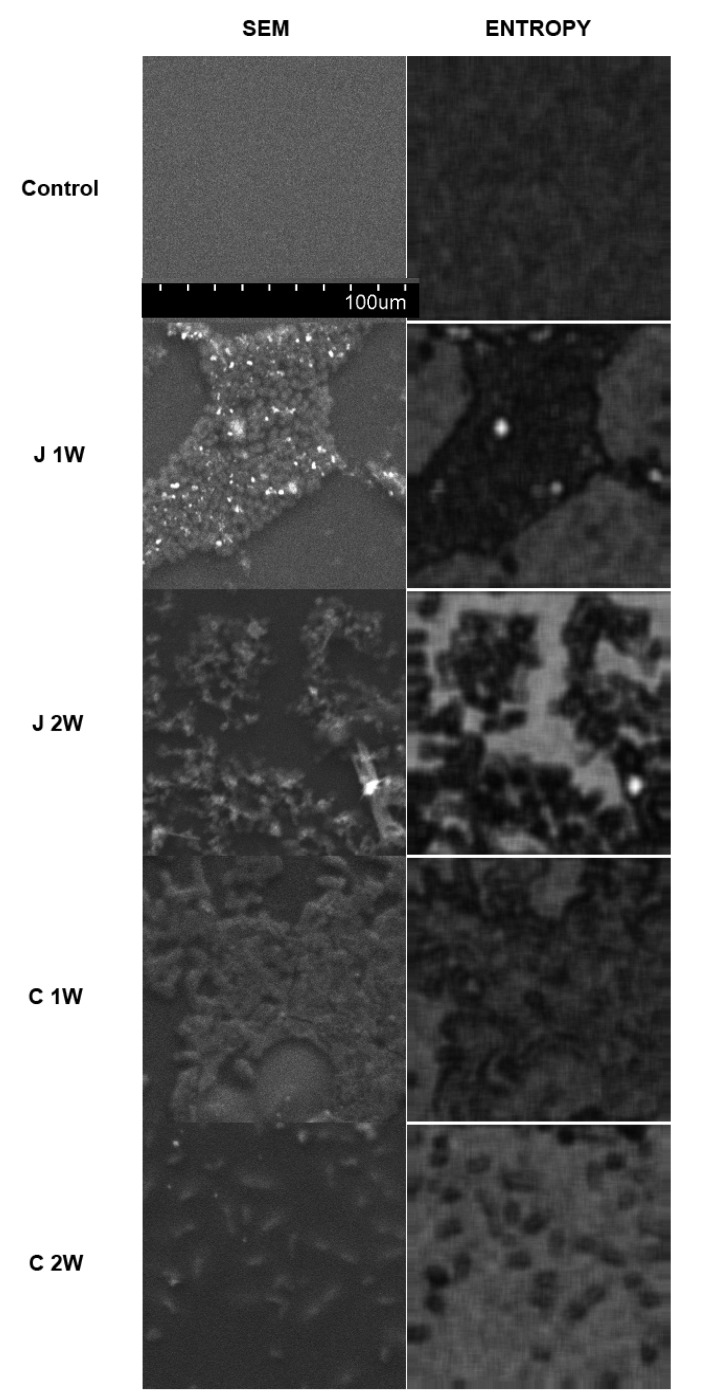
Investigation of surface structures on orthodontic alignments using digital texture analysis. The left column shows images from scanning emission microscopy (SEM). The right column shows intensity maps of chaotic and nonorganized structures (Entropy)—the brighter the local areas, the higher the entropy of the site. In these maps, textures with organized structure have low entropy (dark fields) because their structure is not chaotic.

The average entropy of the aligner surface texture (control) is high and indicates the homogeneity of the material structure and the chaotic arrangement of small elements in the SEM image of the surface. Incubation with the two test substances increases the surface complexity in a time-dependent manner and directly proportional to the time elapsed. After 1 week, visible texture-organized fields appear. These are globular and rod-shaped structures on part of the homogeneous native surface, and after 2 weeks, they cover most of the surface. These image elements are fragmented, which raise the calculated entropy in the samples—Figure 2.

The highest entropy measured was in the control group (3.21 ± 0.03). The next highest was in the C1W group (3.07 ± 0.06, but statistically significantly lower than the control). It was lower in group J1W (2.91 ± 0.06 statistically lower than in control and C1W), and the lowest texture entropy values were in both groups after two weeks of incubation, i.e., C2W and J2W (2.65 ± 0.24 and 2.65 ± 0.23, respectively, significantly lower than the other three groups, *p* < 0.001) (Table 3 and Table 4).

**Figure 3:**
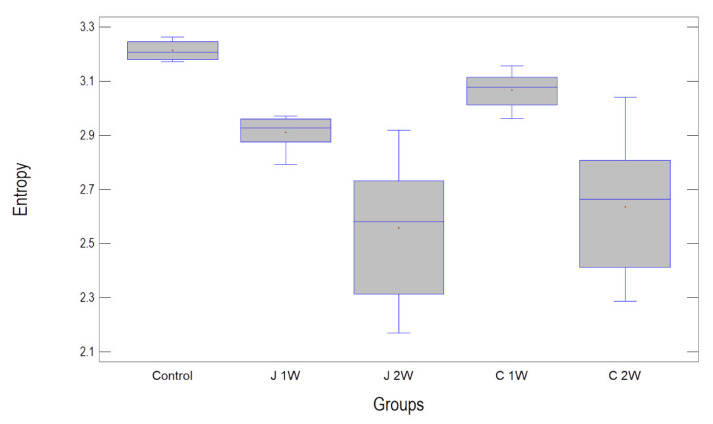
Entropy measured in presented texture on the surface of orthodontic aligners. Contact with incubation fluid decreases entropy in a time-dependent manner. Abbreviations: J1W—orange juice incubation 1 week, J2W—orange juice incubation 2 weeks, C1W—Coca-Cola incubation 1 week, C2W—Coca-Cola incubation 2 weeks.

It should also be noted that there are differences between the two experimental groups. Both after 1 week of incubation (*p* < 0.001) and after 2 weeks (*p* < 0.001), values in group C are higher than in group J—Figure 3.

### 3.3. Wetting Angle

The study aimed to determine the values of the wetting angle and surface tension of samples of the material used to make Clear Aligner braces. The results obtained are shown in the tables below. They were grouped into successive wetting angle results using water and ethyl glycol. Statistically significant differences were found only between the control group and the C1W group for water, as well as between the control group and the J2W and C1W groups and between the J1W and C1W groups for glycol–Table 5.

From the individual measurement results obtained, a slight reduction in the wetting angle values was observed when both solutions—water and the organic solution ethyl glycol—were used for samples exposed to a particular environment, i.e., juice or in a Coca-Cola-type drink. Moreover, a clear difference in angle values between the solutions themselves was observed. Wetting angles were smaller when ethyl glycol was used. However, this is characteristic of organic solutions and this type of measurement. 

The next step of the measurements was to obtain the individual surface tension and total tension values (Table 6 and Table 7). In this case, an increase in surface tension can be seen with a change in the conditions/environment in which the sample was exposed. This increase is evident for both water and ethyl glycol that have been used. Nevertheless, the results for water show a statistically significant difference only between the control group and the C1W group. The other measurements do not show any statistically significant differences.

The wetting angle values determined for the control samples correspond to wettable materials exhibiting more hydrophilic properties (θ < 90°). However, when all results are compared, a clear reduction in wetting angles is observed for samples additionally exposed to a particular solution. The lower the value of the wetting angle, the better the hydrophilic properties and the worse the hydrophobic properties. This is most evident in the surfaces of the samples that have been exposed for a week in the Coca-Cola environment.

## 4. Discussion

Texture analysis was the research method that revealed surface texture changes in all cases as compared to the control group. Statistical analysis showed differences between all groups for this method, except for the C2W/J2W groups. What is notable about these two groups is the extent of the standard deviation, which is an order of magnitude higher than for all of the other tested groups. 

The observed structures on the originally smooth (control) surface of the orthorhombic material disturb the random texture of small elements of an image. Therefore, entropy decreases in experimental groups. This means the appearance of some organized surface structures [23,24]. The sites remaining intact by this process continue to have an underlying high entropy, while the sites of altered texture are significantly organized into globular and elongated formations. This significantly reduces the magnitude of the measured level of texture chaoticity (entropy) [25,26].

The results show that the texture analysis can detect and distinguish changes occurring on the surface of aligners under the influence of foods that were active over a period of one or two weeks. It can be seen that the effect of Coca-Cola for just 1 week led to a change in the surface structure of aligner material. The effect of orange juice was even more pronounced in the same time frame. The action of both beverages over a 2-week period caused a very significant change in the surface entropy of the test elements, but the level of variation in these changes also increased so substantially that, as a consequence, statistical analysis did not yield a statistically significant result when comparing the J2W/C2W groups. 

In conclusion, it can be assumed that the results obtained are in agreement with studies by Kumar et al., Filho et al., and Lombardo et al., who showed that drinks such as Coca-Cola and orange juice can affect the structure of orthodontic materials such as elastomeric chains or aligners [27,28,29].

The results of the analysis indicate that crystallized precipitates from the fluids in which the test samples were immersed may be responsible for some of the changes occurring on the surface of the tested elements. It is important to consider the extent to which such precipitates can contribute to the development of bacterial colonies and biofilms. Another important consideration is whether it is possible to prevent the development of such a biofilm, as is attempted, for example, by modifying the surface of orthodontic wires [30,31].

Although many researchers consider wetting angle goniometry to be a strictly qualitative technique, it can be used to measure certain properties quantitatively. The goniometer allows the contact point at the phase contact to be observed at high magnification, with the wetting angle value and the surface tension value to be obtained as numerical values. 

A comparison of the change in water wetting angle between groups showed a statistically significant difference only for the control/C1W groups. It can be assumed that, in fact, just 1 week of exposure to Coca-Cola on the surface of polymeric orthodontic materials can cause a change in surface texture to occur, but in the light of both the above-discussed texture analysis and studies by other authors [27,28,29], it should be assumed that this is all the more likely to occur with the other groups. The results obtained by the authors should therefore rather lead to the conclusion that the mentioned test method should not be considered reliable for the analysis of aligner surface changes. 

A different situation is found in the analysis of changes in glycol wetting angle. For this measurement, it was again shown that the greatest change occurred when the surface of the aligners was exposed to Coca-Cola for 1 week (control/C1W groups). Nevertheless, a statistically significant difference also occurred between the control/J2W and J1W/C1W groups. Interestingly, the C2W group showed no statistically significant differences from either group. 

Overall, the wetting angle values determined for the control samples correspond to wettable materials exhibiting more hydrophilic properties (θ < 90°). However, when all results are compared, a reduction in wetting angles is observed for samples additionally exposed to a particular solution. The lower the value of the wetting angle, the better the hydrophilic properties and the worse the hydrophobic properties. This is most evident in the surfaces of the samples that have been exposed for a week in Coca-Cola. However, the change in wetting angle did not show a clear time dependence. That is, in the case of Coca-Cola, the decrease in wetting angle was distinctly greater after one week of immersion and then increased but to a value lower than the baseline value. In the case of juice, the wetting angle decreased with increased immersion time. However, the analysis of the wetting angle with water as well as with glycol did not allow the researchers to establish an unambiguous relationship between immersion time and the degree of change, but it is only a confirmation that immersion in orange juice and more significantly in Coca-Cola contributes to the surface degradation of the tested elements. To some extent, this is consistent with the results obtained by the authors in previous studies, in which they found that the oral environment can significantly degrade even metal components used in this environment [32,33]. Beverages that significantly lower the pH of the oral environment are considered to be one of the elements of such an environment [34,35]. Interestingly, virtually no difference was shown in terms of surface tension changes, regardless of the times of immersion in juice and Coca-Cola. 

The most consistent results were obtained when analyzing the fractal dimension changes of the samples tested. Only the C1W group did not show statistically significant differences from any of the analyzed groups. The control group showed a significant difference compared to both groups of samples immersed for 2 weeks (control/C2W and control/J2W). At the same time, a similar variation occurred when comparing the J1W group—immersion for 1 week in juice did not induce changes that would be significantly different compared to the control group, but prolonged exposure to both fluids induced statistically significant changes also in comparison to this group (differences occurred for J1W/J2W and J1W/C2W). 

In general, it can be concluded that cola causes a slightly greater change in fractal dimension value, but the decisive factor for this parameter is time—both liquids strongly changed the values of the fractal dimension after immersion for two weeks only. It should be noted that these have resulted in a reduction in the value of this parameter, thus changing the nature of the disturbance of the homogeneity of the surface from a surface disturbance toward a linear change. This may suggest the formation of micro-cracks on the surface of the aligners as a result of prolonged exposure to both Coca-Cola and orange juice. The occurrence of this type of degradation can alter the mechanical properties of the material and promote damage during use. However, what is significant is that such changes occurred after 2 weeks of immersion. According to recommendations, the aligners should be replaced every two weeks. Some vendors, depending on the type of aligner, recommend replacing it every 1 week. In this case, the obtained results suggest that in the assumed period, there should be no significant changes in the structure of the aligner surface.

Unfortunately, this is not confirmed by an analysis of the occurrence of correlations between the wetting angle of both water and glycol and FD and TA. Each of the measurements: FD, TA, and wetting angle, showed changes that depended on the immersion of the aligners in juice and Coca-Cola. However, the intensity and distribution of these changes varied and did not show statistical significance in terms of correlation analysis. Therefore, it is only possible to descriptively state the direction of change, i.e., the gradual degradation of the surface of the aligners, during immersion in juice and Coca-Cola. 

The results obtained are consistent with a study by Daniele et al. who also showed degradation and a change in the mechanical properties of aligners after 2 weeks of use [36]. The presented results are also in agreement with the findings reported by Paradowska et al. who found that aligner materials, especially after a period of use, promote microbial proliferation [9]. 

### Limitations of the Study

In the presented study, the most popular beverages were used for immersion. The intention of the authors was to show certain tendencies while maintaining maximum clarity. Of course, such a study could be extended to more liquids, and then the results might be more comprehensive. This could make the study more difficult to correctly interpret, however.

The authors resigned from carrying out other analyses of mechanical properties, such as bending tests, for example. In the authors’ opinion, this topic has already been extensively described in the literature. However, the relationship between changes in the surface characteristics studied with the help of three methods and changes in the characteristics of other mechanical properties has not yet been investigated. This could be the subject of a future study.

## 5. Conclusions

Analysis of fractal dimension values showed a clear dependence of the intensity of aligner surface changes on the time of immersion of these elements in Coca-Cola and orange juice.

The texture analysis showed high sensitivity to the changes occurring on the surface of the aligners under immersion in Coca-Cola and orange juice. This analysis, however, was not able to show the intensity of the changes in relation to immersion time.

The measurement of the wetting angle proved that immersion of the aligners in Coca-Cola leads to the degradation of their surface, but it did not prove the dependence of the intensity of this degradation as a function of time.

Repeated immersion in Coca-Cola and orange juice can cause degradation of the surface of the aligners and the formation of microcracks, which supports the recommendation to replace these elements no later than after 2 weeks.

On the surface of the aligners, microprecipitates from beverages such as Coca-Cola or orange juice can be formed. Therefore, it would be appropriate to consider conducting studies to assess their impact on the development of bacterial biofilm on the surface of aligners and possibly develop a method to prevent the deposition of these precipitates or the development of bacterial biofilm.

## Figures and Tables

**Table 1:** Summary statistic of fractal dimension value, and results of Kruskal–Wallis multiple comparison test. (Control—control group, J1W—juice one week, J2W—juice two weeks, C1W—Coca-Cola one week, C2W—Coca-Cola two weeks, n.s.—no statistically significant difference).

Group Number	Name	Count	Average	Median	Standard Deviation	Minimum	Maximum	*p* < 0.05
1	Control	15	1.8032	1.8364	0.1176	1.5118	1.9436	vs. 3, vs. 5
2	J1W	15	1.8400	1.8957	0.1374	1.4986	1.9803	vs. 3, vs. 5
3	J2W	15	1.5944	1.6528	0.1605	1.3031	1.7667	vs. 1, vs. 2
4	C1W	15	1.7615	1.7695	0.1156	1.5016	1.8970	n.s.
5	C2W	15	1.5424	1.5225	0.2560	1.1085	1.8737	vs. 1, vs. 2

**Table 2:** Value of R (Spearman) coefficient between examined variables, FDA—fractal dimension analysis, and TA—texture analysis.

			R Spearman	*p* Value
FDA	vs.	Water wetting angle	0.5000	0.3910
FDA	vs.	Glycol wetting angle	0.5000	0.3910
FDA	vs.	Water surface tension	−0.3000	0.6238
FDA	vs.	Glycol surface tension	−0.1000	0.8729
FDA	vs.	Total tension	−0.6668	0.2189
TA	vs.	Water wetting angle	0.4000	0.5046
TA	vs.	Glycol wetting angle	0.4000	0.5046
TA	vs.	Water surface tension	−0.1000	0.8729
TA	vs.	Glycol surface tension	0.0000	1.0000
TA	vs.	Total tension	−0.8721	0.0539
TA	vs.	FDA	0.6000	0.2848

**Table 3:** Summary statistics for entropy.

Name	Count	Average	Median	Standard Deviation	Minimum	Maximum
Control	15	3.21389	3.20668	0.0333161	3.17229	3.26219
J1W	15	2.91045	2.92633	0.0586776	2.7919	2.97064
J2W	15	2.55829	2.58065	0.233394	2.17102	2.9178
C1W	15	3.06823	3.07739	0.0563168	2.96178	3.15617
C2W	15	2.63535	2.6641	0.237663	2.28786	3.03994

**Table 4:** Statistically significant differences in entropy between groups (* denotes a statistically significant difference).

Contrast	Sig.	Difference	+/− Limits
Control–J1W	*	0.303439	0.112201
Control–J2W	*	0.655598	0.112201
Control–C1W	*	0.145663	0.112201
Control–C2W	*	0.57854	0.112201
J1W–J2W	*	0.352159	0.112201
J1W–C1W	*	−0.157776	0.112201
J1W–C2W	*	0.275101	0.112201
J2W–C1W	*	−0.509935	0.112201
J2W–C2W		−0.0770581	0.112201
C1W–C2W	*	0.432877	0.112201

**Table 5:** Summary statistics of water and glycol wetting angle, and results of Kruskal–Wallis multiple comparison test. (Control—control group, J1W—juice one week, J2W—juice two weeks, C1W—Coca-Cola one week, C2W—Coca-Cola two weeks, and n.s.—no statistically significant difference).

Wetting Angle [°]—Water								
Group Number	Name	Count	Average	Median	Standard Deviation	Minimum	Maximum	*p* < 0.05
1	Control	15	68.9	65.5	5.4	64.2	75.9	vs. 4
2	J1W	15	57.1	53.9	6.8	50.1	64.5	n.s.
3	J2W	15	53.4	52.3	2.7	50.7	57.7	n.s.
4	C1W	15	30.5	36.8	9.9	19.5	38.7	vs. 1
5	C2W	15	53.9	54.6	4.8	48.8	60.7	n.s.
**Wetting Angle [°]—glycol**								
**Group Number**	**Name**	**Count**	**Average**	**Median**	**Standard deviation**	**Minimum**	**Maximum**	***p* < 0.05**
1	Control	15	54.4	54.2	2.0	52.6	57.5	vs. 3, vs. 4
2	J1W	15	44.9	43.5	3.3	42.0	50.3	vs. 4
3	J2W	15	36.1	35.0	2.3	34.1	39.2	vs. 1
4	C1W	15	26.5	26.1	2.4	23.4	29.8	vs. 1, vs. 2
5	C2W	15	42.4	42.7	5.8	35.8	51.2	n.s.

**Table 6:** Summary statistics of water and glycol surface tension, and results of Kruskal–Wallis multiple comparison test. (Control—control group, J1W—juice one week, J2W—juice two weeks, C1W—Coca-Cola one week, C2W—Coca-Cola two weeks, and n.s.—no statistically significant difference).

Surface Tension [mN/m]–Water								
Group Number	Name	Count	Average	Median	Standard Deviation	Minimum	Maximum	*p* < 0.05
1	Control	15	24.9	28.17	9.7	13.4	33.6	4
2	J1W	15	39.4	45.3	13.8	22.2	52.6	n.s.
3	J2W	15	38.8	41.3	7.9	28.0	48.7	n.s.
4	C1W	15	70.9	62.9	13.5	58.0	87.0	1
5	C2W	15	42.2	48.0	11.0	24.1	50.0	n.s.
**Surface Tension [mN/m]–Glycol**								
**Group Number**	**Name**	**Count**	**Average**	**Median**	**Standard Deviation**	**Minimum**	**Maximum**	***p*** **< 0.05**
1	Control	15	10.1	7.8	5.8	4.7	16.9	n.s.
2	J1W	15	6.5	5.0	4.3	2.5	11.9	n.s.
3	J2W	15	9.5	8.5	3.8	5.4	15.0	n.s.
4	C1W	15	2.8	3.1	1.8	0.7	5.3	n.s.
5	C2W	15	7.1	5.4	5.7	2.4	16.8	n.s.

**Table 7:** Summary statistics of total surface tension, and results of Kruskal–Wallis multiple comparison test. (Control—control group, J1W—juice one week, J2W—juice two weeks, C1W—Coca-Cola one week, C2W—Coca-Cola two weeks, and n.s.—no statistically significant difference).

Total Surface Tension [mN/m]								
Group Number	Groups	Count	Average	Median	Standard Deviation	Minimum	Maximum	*p* < 0.05
1	Control	15	35.2	37.0	4.1	30.2	39.0	n.s.
2	J1W	15	47.2	50.3	7.9	37.9	55.5	n.s.
3	J2W	15	48.3	48.9	4.2	42.9	54.1	n.s.
4	C1W	15	35.2	37.0	4.1	30.2	39.0	n.s.
5	C2W	15	49.3	51.9	5.6	40.9	54.0	n.s.

## Data Availability

Data available from the corresponding author upon request.
